# A blood-based metabolomics test to distinguish relapsing–remitting and secondary progressive multiple sclerosis: addressing practical considerations for clinical application

**DOI:** 10.1038/s41598-020-69119-3

**Published:** 2020-07-24

**Authors:** Tianrong Yeo, Megan Sealey, Yifan Zhou, Luisa Saldana, Samantha Loveless, Timothy D. W. Claridge, Neil Robertson, Gabriele DeLuca, Jacqueline Palace, Daniel C. Anthony, Fay Probert

**Affiliations:** 1 Department of Pharmacology, University of Oxford, Mansfield Road, Oxford, OX1 3QT UK; 2 Department of Neurology, National Neuroscience Institute, 11 Jalan Tan Tock Seng, Singapore, 308433 Singapore; 3 Nuffield Department of Clinical Neurosciences, Level 6, West Wing, John Radcliffe Hospital, University of Oxford, Headley Way, Oxford, OX3 9DU UK; 4 Division of Psychological Medicine and Clinical Neuroscience, School of Medicine, University Hospital of Wales, Cardiff University, Cardiff, CF14 4XN UK; 5 Chemistry Research Laboratory, Department of Chemistry, University of Oxford, Mansfield Road, Oxford, OX1 3TA UK

**Keywords:** Multiple sclerosis, Metabolomics

## Abstract

The transition from relapsing–remitting multiple sclerosis (RRMS) to secondary progressive MS (SPMS) represents a huge clinical challenge. We previously demonstrated that serum metabolomics could distinguish RRMS from SPMS with high diagnostic accuracy. As differing sample-handling protocols can affect the blood metabolite profile, it is vital to understand which factors may influence the accuracy of this metabolomics-based test in a clinical setting. Herein, we aim to further validate the high accuracy of this metabolomics test and to determine if this is maintained in a ‘real-life’ clinical environment. Blood from 31 RRMS and 28 SPMS patients was subjected to different sample-handling protocols representing variations encountered in clinics. The effect of freeze–thaw cycles (0 or 1) and time to erythrocyte removal (30, 120, or 240 min) on the accuracy of the test was investigated. For test development, samples from the *optimised* protocol (30 min standing time, 0 freeze–thaw) were used, resulting in high diagnostic accuracy (mean ± SD, 91.0 ± 3.0%). This test remained able to discriminate RRMS and SPMS samples that had experienced additional freeze–thaw, and increased standing times of 120 and 240 min with accuracies ranging from 85.5 to 88.0%, because the top discriminatory metabolite biomarkers from the *optimised* protocol remained discriminatory between RRMS and SPMS despite these sample-handling variations. In conclusion, while strict sample-handling is essential for the development of metabolomics-based blood tests, the results confirmed that the RRMS vs. SPMS test is resistant to sample-handling variations and can distinguish these two MS stages in the clinics.

## Introduction

The clinical transition from relapsing–remitting multiple sclerosis (RRMS) to secondary progressive MS (SPMS) represents a diagnostic challenge as progression is typically gradual and objective clinical signs often lag behind patient’s symptoms^1^. While inflammation and neurodegeneration can occur continuously, clinically defined secondary progression often occurs in a non-uniform manner^2^, whereby progression is interspersed with periods of relative clinical stability. There are currently no validated biofluid or imaging biomarkers that can reliably separate these two stages of MS^3^ and, as a result, SPMS diagnosis can often only be made after observing progression of disability over a prolonged period of time, in some cases, years. As such, the diagnosis of SPMS is always established retrospectively when an irreversible accrual of disability has already occurred^3^.

Metabolomics is an emerging approach for biomarker discovery in precision medicine and for identifying disease pathways underpinning clinical phenotypes^4,5^. Metabolomics involves the comprehensive study of the metabolome; all low molecular weight (< 1,500 Da) metabolites within a biological sample. As metabolites are the biological end products of upstream processes involving gene and protein expression, the metabolome closely reflects the clinical phenotype and, thus, can provide valuable insight in to underlying pathological processes and identify novel biomarkers of disease. The majority of metabolomics studies in MS focus on the identification of blood-borne metabolite perturbations in MS patients relative to healthy controls using both nuclear magnetic resonance (NMR)-based^6–8^ and mass spectrometry-based metabolomics^9–12^. While this is useful in aiding our understanding of MS disease activity as a whole, the distinction between MS and controls is not a clinical diagnostic challenge. In contrast, relatively few studies have investigated the metabolite changes associated with the transition from RRMS to SPMS. We were the first to show, using serum ^1^H NMR metabolomics, that RRMS and SPMS can be differentiated^13^ and highlighted the potential of this technique to objectively distinguish between these two phases of MS. Other studies have since followed suit, further validating the presence of distinct metabolic changes in SPMS relative to RRMS in cerebrospinal fluid (CSF) using mass spectrometry methods^15^. In addition, a targeted mass spectrometry-based metabolomics study discovered that serum quinolinic acid levels were highest in primary progressive (PP) MS patients and decreased in SPMS and then RRMS patients respectively^16^. Other studies have investigated both PPMS and SPMS patients to determine biomarkers in progressive disease, as a whole, relative to RRMS. In blood, perturbations in energy metabolism have been observed using HPLC metabolomics methods. While the metabolite changes identified correlated with EDSS and MRI measures of neurodegeneration, their diagnostic accuracy is not known^17,18^. In CSF, a panel of 250 proteins has been identified which is able to distinguish between RRMS and progressive (SPMS and PPMS combined) MS with an accuracy of 89.4% in a validation cohort^19^. While such a test could be useful, monitoring of the transition from RRMS to SPMS in a clinical setting may be challenging due to the high cost of measuring large panels of proteins coupled with the invasive nature of CSF sampling. While our previous results coupled with the above reports provide evidence for the potential of metabolomics analysis in the diagnosis of MS, no studies have systematically investigated the tolerances of such diagnostic models in common clinical settings.

It is well known that metabolic alterations within blood samples can occur due to variations in pre-analytical sample-handling^20–26^. As a result, stringent processing and storage protocols are implemented in the initial (research) phase of metabolomics blood test development^27^, which can be difficult to follow in clinical practice. While metabolic perturbations introduced by freeze–thaw and delayed centrifugation have been previously described in healthy individuals^20,21,23,24,26,28^, there is a paucity of studies addressing how these factors may affect the accuracy of a diagnostic metabolomics test in a clinical environment. Indeed, the extent to which sample-handling variation affects overall diagnostic accuracy is likely to be disease and application specific, and dependent on the stability of the discriminatory metabolites identified. We have observed that at the John Radcliffe Hospital (Oxford, UK), samples were centrifuged within 4 h of collection with an average of 50 min. Therefore, to ensure that our RRMS *vs.* SPMS diagnostic test is applicable in a clinical setting, it is vital that the high diagnostic accuracy is maintained even in instances when the sample-handling protocol may vary. In this cross-sectional study, we further validate our metabolomics diagnostic test on an independent, prospective and well-characterised set of RRMS and SPMS samples (using the sample-handling protocol currently well-accepted in metabolomics) and investigate the impact of two of the most common sources of variation in sample-handling identified in our clinic: (1) freeze–thaw, and (2) delayed centrifugation.

## Methods

### Subjects

Thirty-one RRMS patients and 28 SPMS patients were prospectively recruited from the Oxford University Hospitals Trust from November 2017 to July 2018. All patients recruited at Oxford were consented under the Oxford Radcliffe Biobank, approved by the NRES Committee South Central—Oxford C (REC reference: 09/H0606/5+5), and all research was performed in accordance with relevant guidelines and regulations.

In order, to determine the effect of long-term storage on metabolite concentrations, serum samples were requested from a cohort of 30 RRMS and 50 SPMS patients from the Welsh Neuroscience Research Tissue Bank, Cardiff University (REC reference: 19/WA/0058). These samples were stored at − 80 °C for between 1 and 10 years. Patient information for this, additional, cohort can be found in Table S2.

All patients fulfilled the 2017 revisions to the McDonald criteria for MS^29^. SPMS status was established clinically by MS neurologists; all SPMS patients demonstrated progressive accrual of disability over at least 1 year independent of relapses^30^, and had Expanded Disability Status Scale (EDSS) ≥ 4.5 at the time of confirmed disability progression^31,32^. Clinical and demographic data were obtained from medical notes and patient interviews. Current EDSS was assessed on the day of recruitment, prior to blood sampling.

### Blood collection, serum processing and NMR sample preparation

Blood was collected in BD Vacutainer tubes (BD 367837). The *optimised* sample-handling protocol is used frequently in metabolomics literature and involved the following steps^26,33^: once collected, blood was left to stand for 30 min at room temperature; blood was then centrifuged at 1,300×*g* for 10 min at room temperature for erythrocyte separation to obtain serum; serum was then immediately aliquoted and stored at − 80 °C until NMR sample preparation. For NMR sample preparation, serum was thawed at room temperature followed by ultra-centrifugation at 100,000×*g* for 30 min at 4 °C. 150 μL of the supernatant was then diluted with 400 μL of 75 mM sodium phosphate buffer prepared in D_2_O (pH 7.4) and stored at − 80 °C until NMR analysis. Immediately before NMR analysis, the buffered NMR sample was thawed at room temperature and then transferred to a 5 mm borosilicate glass tube (Norell 502-7).

### Variations of the optimised protocol

**Figure 1:**
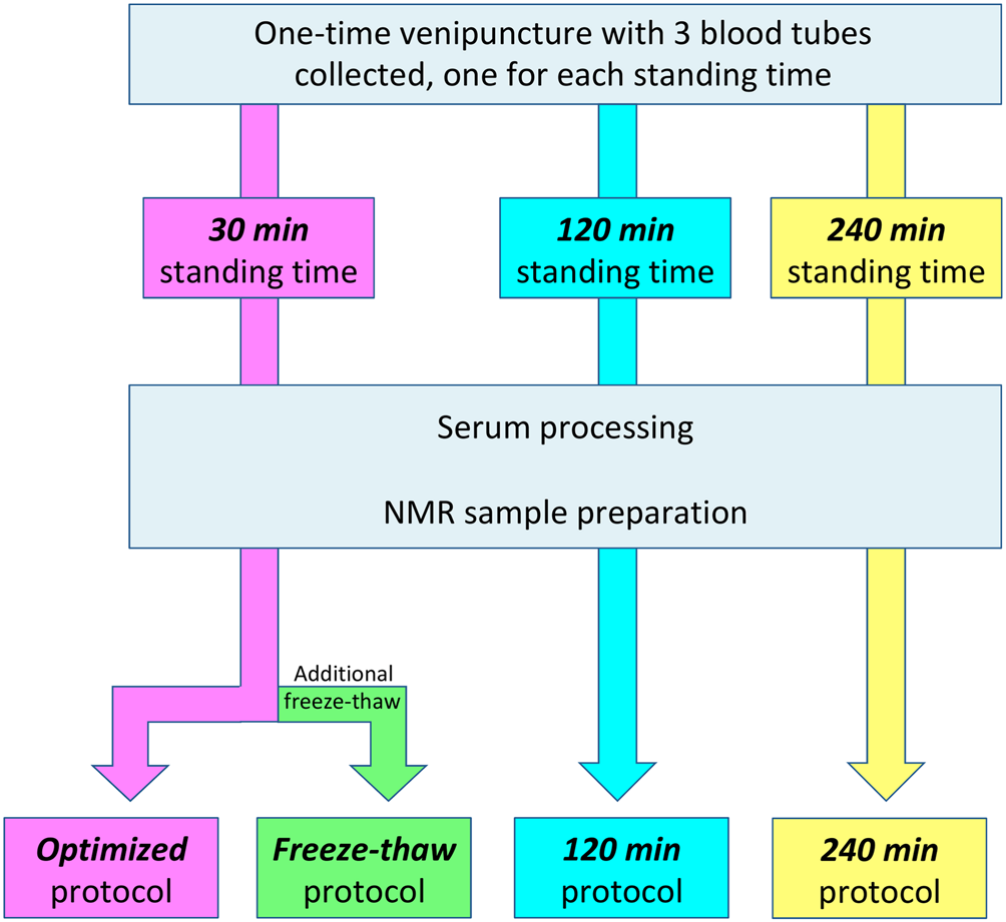
Flow diagram illustrating the sample-handling protocols investigated. The effect of freeze–thaw and increased standing time were investigated and compared to the ideal, *optimised* (30 min) protocol. *Min* minutes, *NMR* nuclear magnetic resonance.

Three variations to the *optimised* protocol were introduced to resemble practical considerations encountered in the clinic and laboratory (Fig. 1). The *freeze–thaw* protocol was identical to the *optimised* protocol, but with an additional freeze–thaw to simulate a scenario whereby the NMR sample has already been prepared, but the NMR spectrometer was unavailable due to logistical or technical issues. The *120 *min and *240* min protocols differed from the *optimised* protocol with 120 min and 240 min of standing time after venipuncture respectively before erythrocyte separation. These 2 protocol variations parallel a very common scenario whereby blood has been taken but not centrifuged in a timely manner due to manpower demands in a busy clinic or laboratory. Apart from the protocol variations of interest, all other processes were kept strictly the same.

### ^1^H NMR metabolomics spectra acquisition

All NMR experiments were performed using a 700-MHz Bruker AVIII spectrometer. To reduce the possibility of ‘batch effect’ bias, samples from the four protocols and the different classes (RRMS and SPMS) were mixed/randomized throughout the NMR run and data acquired in a blinded manner. To reduce inter-operator variability, all NMR samples were prepared by the same person. The same batch of reagents was used to prepare all samples. Four quality control samples were included throughout the run (one at the beginning, one at the end, and then at equal intervals throughout) to ensure reproducibility and minimal variation across spectra.

Technical details of the NMR experiments and data handling have been previously published^34^. In brief, 1D ^1^H (NMR) spectra were obtained using a Carr–Purcell–Meiboom–Gill (CPMG) pulse sequence which retains resonances from small molecular weight metabolites and mobile side chains of lipoproteins, while suppressing broad signals arising from large molecular weight serum components. The CPMG spectra were processed in Topspin (version 3.5, Bruker, Germany) followed by visual inspection to ensure precise referencing and baseline correction, and to check for spectral distortion or contamination. The processed spectra were then exported to ACD/Labs Spectrus Processor Academic Edition 12.01 (Advanced Chemistry Development, Inc., Toronto, Canada), whereby regions of the spectra between 0.80 and 4.20 parts per million (ppm) and 5.20–8.50 ppm were split into 0.02 ppm wide ‘bins’. Integral values of these spectral ‘bins’ were computed with sum normalisation and used as quantitative variables expressed in arbitrary units (AU). ‘Bins’ which contained no spectral resonances on visual inspection (i.e. noise) were excluded, as were ‘bins’ with a coefficient of variation exceeding 15% (calculated across the quality control samples). In all, 185 metabolite ‘bins’ were available for supervised multivariate statistical analysis. Metabolite assignments were performed by referencing to literature values and the Human Metabolome Database^35^. Further confirmation was done by inspection of the 2D pre-saturation correlation spectroscopy (COSY) spectra, spiking of known compounds, and 1D total correlation spectroscopy (TOCSY) spectra.

### Univariate statistical analysis

All statistical analysis, apart from spectral analysis (see below), were performed with STATA software (Release 14, College Station, TX: Statacorp LP) and GraphPad Prism (version 6, California, USA). Comparative analyses between RRMS and SPMS patients were performed using Mann–Whitney *U* test or 2-sample t-test as appropriate for continuous variables, and with Chi squared test for categorical variables. Pearson’s or Spearman's correlation was used to explore correlations depending on data normality. Repeated-measures 2-way ANOVA was used to explore potential interactions and Sidak’s test was applied for multiple comparisons. Two-tailed p values < 0.05 were considered statistically significant.

### Multivariate statistical analysis

It should be noted that all OPLS-DA models presented here (whether developed using the optimal or sub-optimal sample-handing protocols) were validated on independent test data using external tenfold cross validation with repetition and permutation testing. No patient was included in both the *training* and *test* datasets simultaneously.

To identify metabolic differences and develop a multivariate diagnostic model between RRMS and SPMS, orthogonal partial-least square discriminant analysis (OPLS-DA) was performed on samples collected using the *optimised* protocol (recommended standard in the metabolomics field)^34^. All OPLS-DA models were thoroughly validated on independent test data using external tenfold cross-validation with repetition using in-house R scripts (R foundation for statistical computing, Vienna, Austria)^36^, and the *ropls* package^37^. Ten-fold external cross-validation with 100 iterations was performed, creating an ensemble of 1,000 models. Further details of this approach have been previously published^34^. In brief, this process involves repeated cycles of: (1) balancing class sizes, (2) random division of the spectral data into a *training* set (90% of data) and a *test* set (remaining 10% of data), (3) construction of OPLS-DA models using the *training* set alone, and (4) determining the predictive accuracy, sensitivity, and specificity of the OPLS-DA model using the independent *test* set. The validity of the metabolic separation between RRMS and SPMS was established if the mean predictive accuracy of the ensemble (1,000 models) of model accuracies was significantly higher compared to the mean predictive accuracy of a separate ensemble (also 1,000 models) created by random class assignments on the same spectral data.

To determine how well the above diagnostic model developed performs on samples that have experienced freeze–thaw or increased standing time, the same external tenfold cross-validation strategy was employed with random subsets of the (1) *freeze–thaw*, (2) *120* min, and (3) *240* min samples selected as *test* sets. This replicates a scenario in which a diagnostic model developed on samples collected in a research setting is applied to samples collected in a clinical setting. No sample from the same patient existed in both the *training* and *test* sets simultaneously; i.e. the *test* set was always independent of the *training* set.

We also investigated whether samples collected with sub-optimal protocols can be used in a research setting to develop multivariate models and accurately identify metabolite biomarkers by using the OPLS-DA strategy described above, by using only samples from the *freeze–thaw*, *120* min, and *240* min protocols (in both *training* and *test* sets). Finally, we investigated whether samples stored long-term (up to 10 years) can be used to identify metabolite biomarkers by applying the same analytical strategy to a cohort of RRMS and SPMS patients from the Welsh Neuroscience Research Tissue Bank, Cardiff University (REC reference: 19/WA/0058). Direct comparison of the diagnostic accuracies and discriminatory metabolites identified by these models against the model developed using the *optimised* protocol provides valuable insight into the advantages and pitfalls of sample-handling variations for metabolomics analysis and biomarker identification.

### Ethics approval and consent to participate

This study was approved by the Oxford Radcliffe Biobank (NRES Committee South Central—Oxford C, REC reference: 09/H0606/5+5) and the Welsh Neuroscience Research Tissue Bank, Cardiff University (REC reference: 19/WA/0058). All patients gave their (written) informed consent to participate in the study.

### Consent for publication

The consent for publication was given by all authors.

## Results

### Clinical characteristics of study cohort

Thirty-one RRMS patients and 28 SPMS patients were recruited into the study. As expected, SPMS patients were older, had longer disease duration, lower annualised relapse rate (ARR) in the previous 2 years, higher EDSS, and none were on disease-modifying therapies (DMT) (Table 1). No differences in gender were observed, with a preponderance of females in both groups.

**Table 1:** Clinical and demographic characteristics of the study cohort.

	RRMS (n = 31)	SPMS (n = 28)	p value
Age (years,) mean ± SD	43.5 ± 9.7	58.1 ± 9.6	< 0.001
Female, no. (%)	23 (74.2)	20 (71.4)	0.811
Disease duration (years), median (range)	9.2 (0.8–24.2)	26.6 (3.9–50.3)	< 0.001
ARR^a^ last 2 years, median (range)	0.0 (0.0–1.5)	0.0 (0.0–0.0)	< 0.001
Time since last relapse (months), median (range)	23.8 (0.92–142.9)	–	–
EDSS, median (range)	2.5 (1.0–6.5)	7.0 (4.5–8.5)	< 0.001
DMT use, no. (%)	20 (64.5)	0 (0.0)	< 0.001
Glatiramer acetate	8 (25.8)	–	–
Interferon beta	2 (6.5)	–	–
Dimethyl fumarate	7 (22.6)	–	–
Fingolimod	1 (3.2)	–	–
Natalizumab	1 (3.2)	–	–
Alemtuzumab	1 (3.2)	–	–
BMI, mean ± SD	25.9 ± 4.6	28.0 ± 5.9	0.132

*ARR* annualised relapse rate, *BMI* body mass index, *DMT* disease modifying therapy, *EDS*S Expanded Disability Status Scale, *RRMS* relapsing–remitting MS, *SD* standard deviation, *SPMS* secondary progressive MS.

^a^ARR calculated only if disease duration is at least 2 years.

### Blood samples from the optimised protocol result in well-validated OPLS-DA models with a mean predictive accuracy of 91.0%

**Figure 2:**
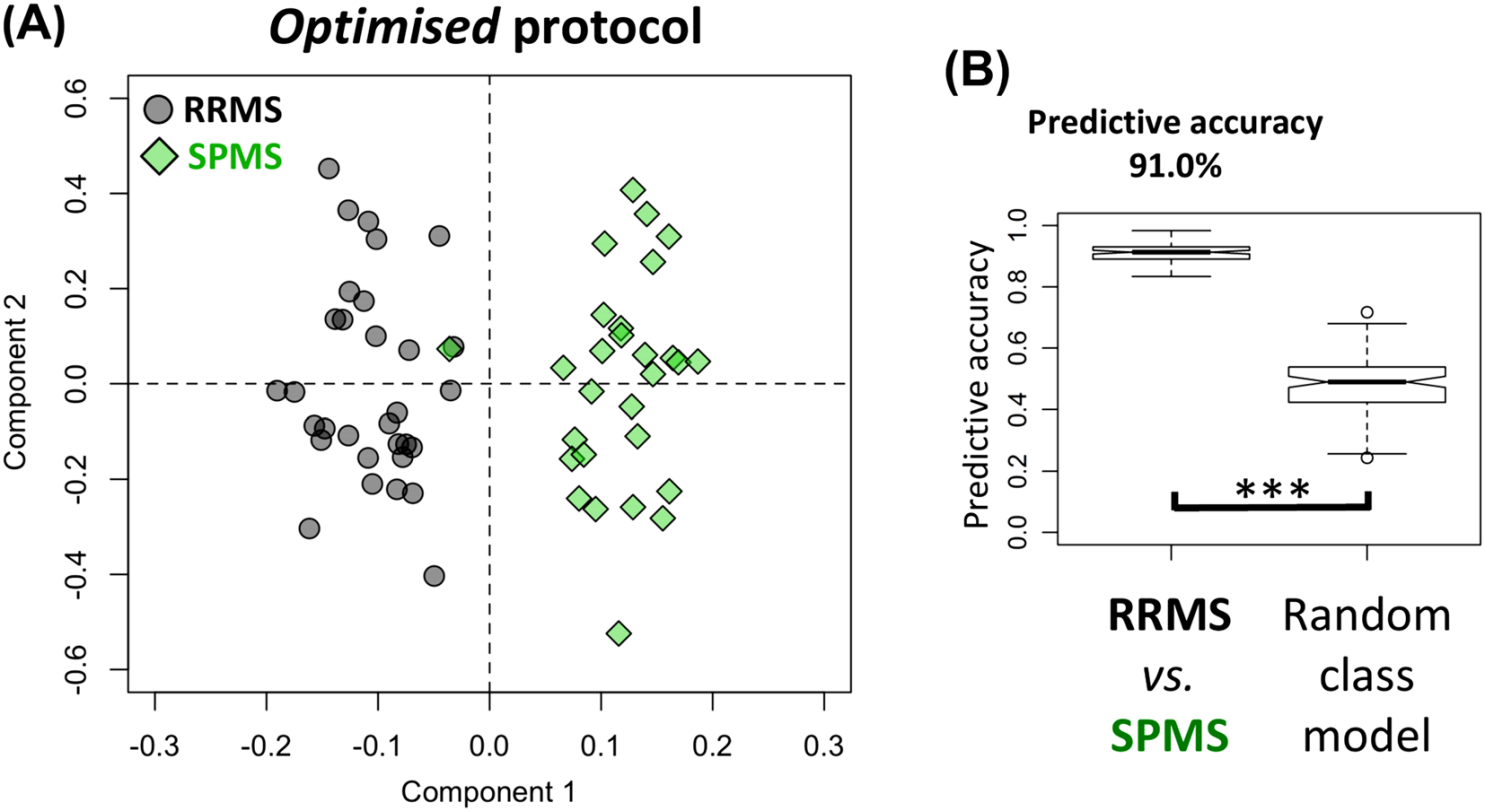
Performance of the OPLS-DA models generated using samples from the *optimised* sample-handling protocol. (**A**) The representative OPLS-DA scores plot of this *well-validated* model illustrates a distinct separation between RRMS and SPMS patients, (**B**) with a high predictive accuracy of 91.0 ± 3.0%. Each point in the scores plot represents all metabolomics spectral data from 1 patient condensed into a single data point. Points (i.e. patients) located closer to one another on the scores plot are metabolically more similar than those further apart. *OPLS-DA* orthogonal partial-least square discriminant analysis, *RRMS* relapsing–remitting MS, *SPMS* secondary progressive MS. Kolmogorov Smirnov test, ***p < 0.001.

In order to validate our previous results using a new independent cohort, samples collected using the *optimised* protocol (recommended protocol in metabolomics literature) were used to construct discriminatory OPLS-DA models to segment RRMS from SPMS (i.e. both *training* and *test* data from the *optimised* protocol). The representative OPLS-DA scores plot showed excellent separation between RRMS and SPMS patients (Fig. 2A). The mean predictive accuracy for the ensemble of the OPLS-DA models of RRMS vs. SPMS was significantly higher than the mean predictive accuracy of the ensemble created by random class assignments (mean ± SD, 91.0 ± 3.0% vs. 47.8 ± 9.5%; p < 0.001) (Fig. 2B). Sensitivity and specificity indices can be found in Table S1. One SPMS patient lies towards the boundary of the RRMS region of the OPLS-DA scores plot (Fig. 2). This patient was diagnosed as SPMS at the time of blood sample collection and her EDSS was 4.5. As a result, it is possible that the positioning of this sample in the scores plot represents the metabolic profile of early progressive disease and, potentially, the transition from RRMS to SPMS. Ongoing work, investigating serial samples of patients transitioning from RRMS to SPMS will elucidate this further.

To identify the most discriminatory metabolites driving the distinction between RRMS and SPMS, variable importance in projection (VIP) scores were generated. The VIP score is a measure of the importance of a metabolite in the OPLS-DA model; the higher the VIP score, the greater the contribution a metabolite makes to the model^37^. Seven metabolites were identified as highly discriminatory; the VIP cutoff of 1.48 was obtained by identifying the inflexion point in the VIP ranking plot (Fig. S1). Details of these 7 discriminatory metabolites are shown in Table 2. In summary, lipoproteins, choline and 3-hydroxybutyrate are lower in SPMS compared to RRMS, while glucose and N-acetylated glycoproteins/glycolipids are higher. These discriminatory metabolites, and indeed their directions, are consistent with observations from our previous cohort^13^, providing strong evidence that the identified metabolites are biomarkers of MS disease stage.

**Table 2:** The identified discriminatory metabolites from the *well-validated* OPLS-DA model.

Discriminatory metabolites	Chemical shift of contributing spectral ‘bins’ (VIP score, VIP rank)	Fold change in SPMS relative to RRMS
Mobile –CH_3_ HDL/LDL	0.80….0.82 ppm (2.15, 8) 0.82….0.84 ppm (3.10, 5) 0.84….0.86 ppm (4.29, 1) 0.86….0.88 ppm (2.52, 6)	0.83
3-Hydroxybutyrate	1.20….1.22 ppm (2.07, 10)	0.84
Mobile (–CH_2_–)n LDL	1.22….1.24 ppm (3.24, 4) 1.24….1.26 ppm (3.46, 3)	0.84
Mobile (–CH_2_–)n Chylomicron/VLDL	1.26….1.28 ppm (2.12, 9)	0.92
NAC1/=CH–CH_2_–CH_2_–	2.04….2.06 ppm (1.48, 12)	1.07
Mobile –N(CH_3_)_3_/free Choline	3.20….3.22 ppm (2.38, 7) 3.22….3.24 ppm (3.69, 2)	0.86
Glucose	3.88….3.90 ppm (1.50, 11)	1.07

*HDL* high density lipoprotein, *LDL* low density lipoprotein, *NAC* N-acetyl-cysteine, *OPLS-DA* orthogonal partial-least square discriminant analysis, *RRMS* relapsing–remitting MS, *SPMS* secondary progressive MS, *VIP* variable importance in projection, *VLDL* very low density lipoprotein.

### Potential confounders do not account for the metabolic separation between RRMS and SPMS

We explored if baseline differences between RRMS and SPMS patients could account for their metabolic separation. Firstly, there were no strong correlations (i.e. all R^2^ < 0.3) of any of the discriminatory metabolites with age, disease duration, number of relapses in the last 2 years, EDSS, body mass index (BMI), time from last meal, or number of units of alcohol consumed per week, and there were no significant associations between any of the metabolites with comorbidity status. Within RRMS patients, there were no differences in any of the metabolite levels stratified by DMT status. Secondly, investigation of the OPLS-DA scores plot revealed no clustering of the potential confounders (expressed as binary variables) confirming no confounding effect (Fig. S2). Importantly, RRMS patients who were ≥ 50 years old or had EDSS ≥ 6.0 did not cluster near to the SPMS patients, and RRMS patients on DMT were spread throughout the RRMS cluster including those nearest to the SPMS cluster. Patients with BMI ≥ 30, consumed ≥ 1 units of alcohol per week, and were fed or fasted were also evenly distributed across both groups. Smoking was not explored as a confounder as there were only 3 current smokers in the entire cohort.

### The well-validated OPLS-DA model is resistant to variations in sample-handling and is still able to stage MS accurately

**Figure 3:**
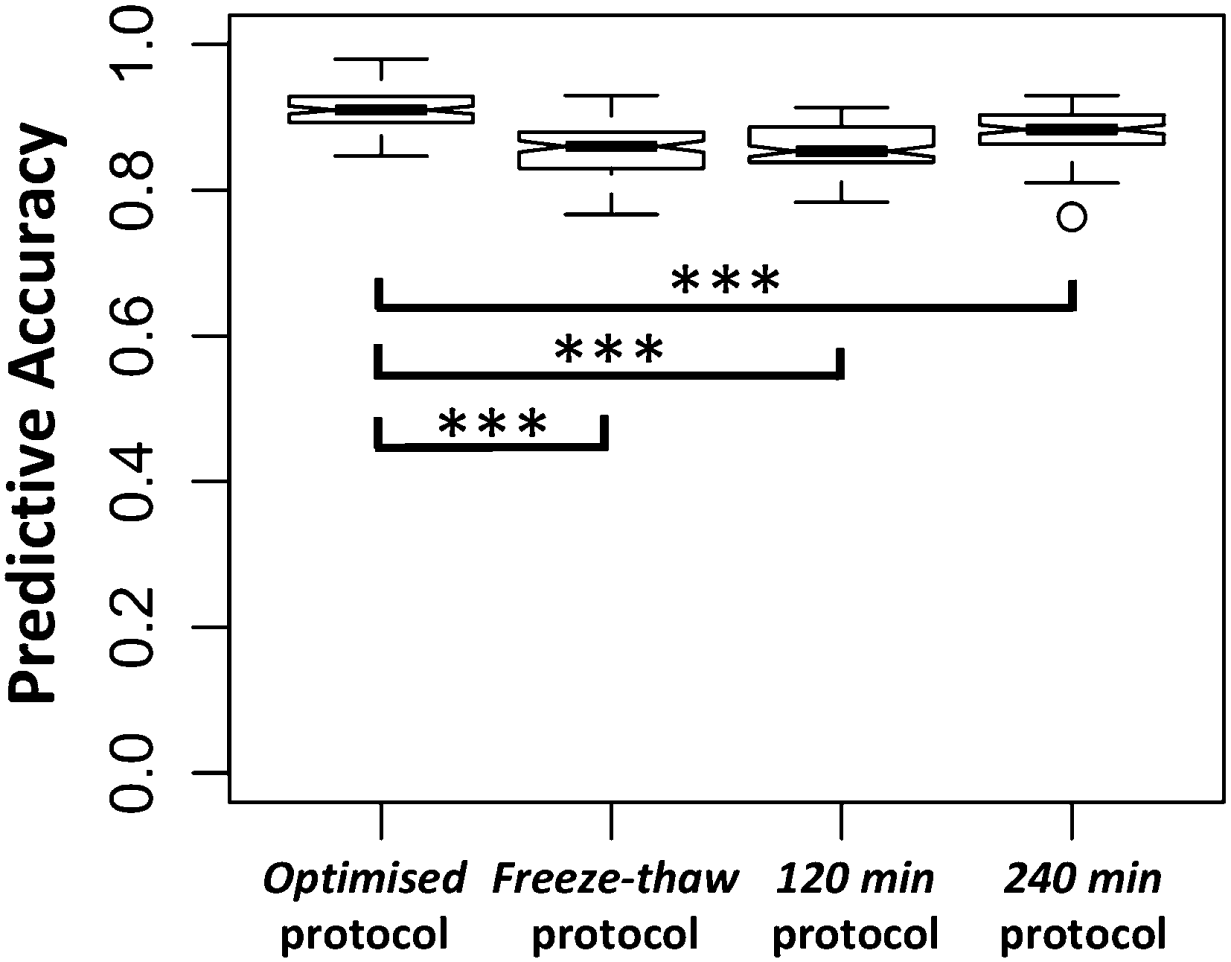
Box plots illustrating the diagnostic accuracy of the *well-validated* discriminatory RRMS *vs.* SPMS OPLS-DA model on sub-optimal samples, which have undergone *freeze–thaw* or increased standing time of *120* min and *240* min. *Min* minutes, *OPLS-DA* orthogonal partial-least square discriminant analysis, *RRMS* relapsing–remitting MS, *SPMS* secondary progressive MS. Comparative analysis was performed using one-way ANOVA with Tukey’s post-hoc corrections. ***p < 0.001.

Next, we explored if the high predictive accuracy of our *well-validated* model (Fig. 2) is maintained when the handling of samples is sub-optimal, i.e. increasing standing time, or an additional freeze–thaw. This is akin to developing a metabolomics blood test using stringent samples (in a research setting) and then applying the test in a clinical setting. The *well-validated* OPLS-DA models were able to predict the diagnosis of samples from the *freeze–thaw*, *120* min, and *240* min protocols with accuracies of 85.5 ± 3.8%, 85.9 ± 3.1%, and 88.0 ± 3.0% respectively (Fig. 3). This translates to a relative accuracy reduction of 6.0%, 5.6% and 3.3% respectively, when compared to using *optimised* protocol samples as the *test* set. Sensitivity and specificity indices can be found in Table S1. Of note, samples left standing for *120* min and *240* min after venipuncture maintained very high specificities for SPMS (94.0% and 93.0% respectively), allowing SPMS to be ‘ruled in’.

### While serum metabolite concentrations vary as a result of sample-handling, the identified metabolite biomarkers remain discriminatory for RRMS and SPMS

**Figure 4:**
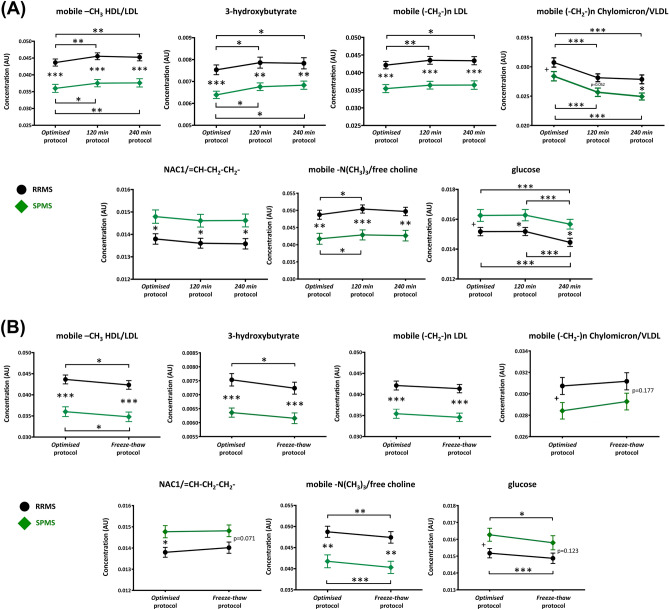
The effect of (**A**) increasing standing time, and (**B**) freeze–thaw on the identified serum metabolite biomarkers distinguishing RRMS (black circle) and SPMS (green diamond). *HDL* high density lipoprotein, *LDL* low density lipoprotein, *Min* minutes, *NAC* N-acetyl-cysteine, *RRMS* relapsing–remitting MS, *SPMS* secondary progressive MS, *VIP* variable importance in projection, *VLDL* very low density lipoprotein. Repeated measures 2-way ANOVA was performed with Sidak’s post-hoc test. *p < 0.05, **p < 0.01, ***p < 0.001. ^**+**^Statistically significant from multivariate OPLS-DA. Error bars represent standard error of the mean.

Next, to explore if the 7 discriminatory metabolites identified by our *well-validated* model (Fig. 2) remained discriminatory with sub-optimal protocols, repeated-measures 2-way ANOVA (the 2 factors being MS subtype and protocol variation) was performed. All metabolites remained discriminatory with variations in standing time (Fig. 4A), while most were still able to distinguish RRMS *vs.* SPMS despite an additional freeze–thaw (Fig. 4B). There was no interaction between MS subtype with any of the protocol variations within all the metabolite biomarkers indicating that the differences in metabolite levels between RRMS and SPMS are similar despite sample-handling variations, and analogous metabolic perturbations occur in RRMS and SPMS sera.

### Using samples collected under sub-optimal protocols for model development reduces predictive accuracies

**Figure 5:**
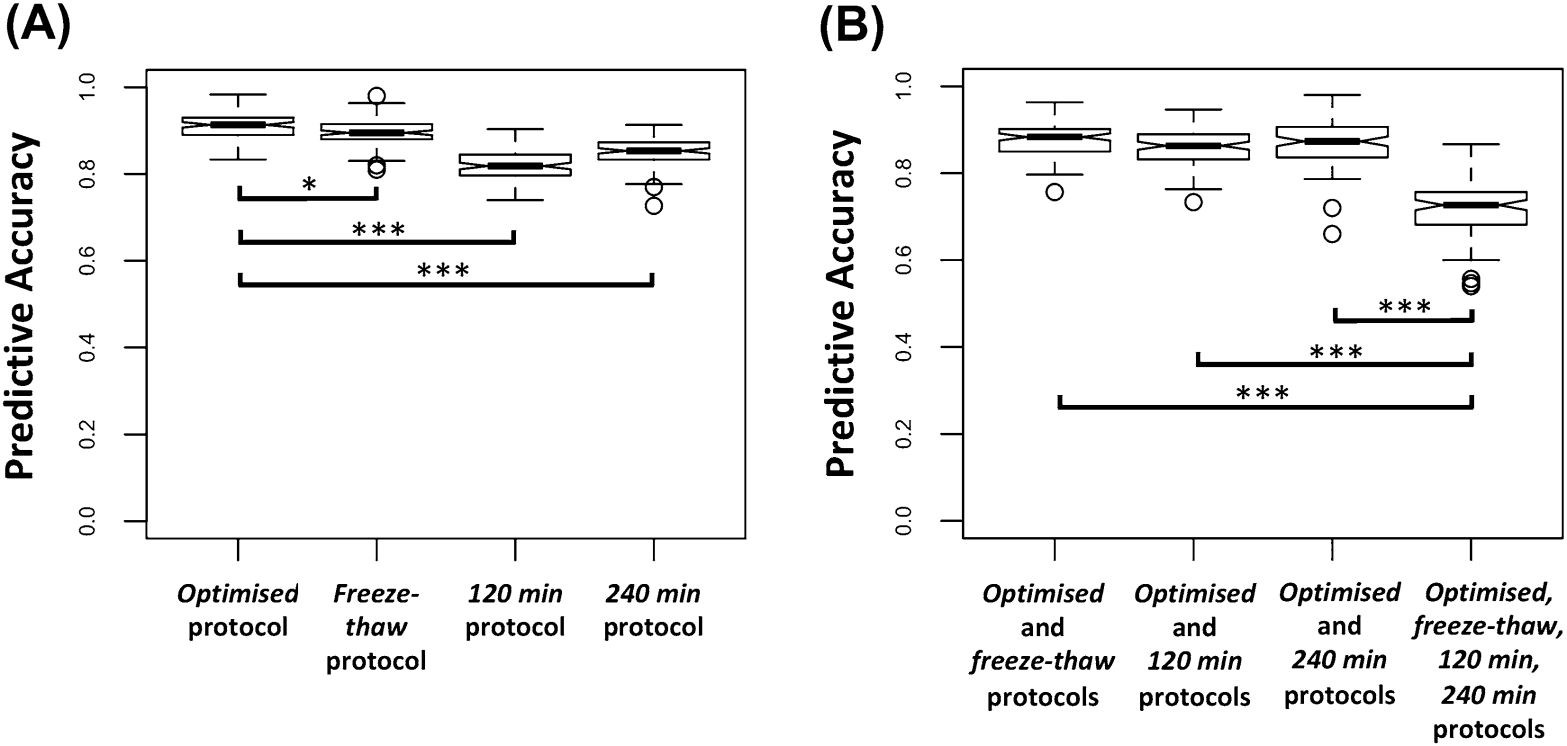
(**A**) Box plots of OPLS-DA model predictive accuracies using samples within each protocol as both the *training* and *test* sets. (**B**) Box plots of OPLS-DA model predictive accuracies using combinations of samples from the *optimised* protocol and from sub-optimal protocols as both the *training* and *test* sets. *Min* minutes, *OPLS-DA* orthogonal partial-least square discriminant analysis. Comparative analysis was performed using one-way ANOVA with Tukey’s post-hoc corrections. *p < 0.05, ***p < 0.001.

Next, we explored if samples from the *optimised* protocol are strictly required for metabolomics test development, or if samples from sub-optimal protocols could be utilised for this purpose. OPLS-DA models developed (and validating by tenfold cross-validation and permutation testing) using samples within the *freeze–thaw*, *120* min and *240* min protocols (as both *training* and *test* sets) resulted in predictive accuracies of 89.6 ± 3.3%, 81.7 ± 3.5% and 84.9 ± 3.2% respectively (Fig. 5A). As the effect of sample-handling conditions on the top discriminatory metabolites was only modest, the top 5 discriminatory metabolites selected by each model were the same (Fig. S3). Due to the significant impact of freeze–thaw on glucose and NAC1 levels, these metabolites were ranked significantly lower in the ‘*freeze–thaw* model’. There was no advantage to using a combination of samples from the *optimised* protocol and sub-optimal protocols (*optimised*/*freeze–thaw, optimised*/*120* min*, optimised*/*240* min*, optimised/freeze–thaw/120* min*/240* min) for test development (Fig. 5B). Indeed, using a combination of all protocols for model development (representing a scenario with highly variable sample-handling) resulted in a significant decrease in diagnostic accuracy to 71.9 ± 6.3%. Sensitivity and specificity indices for all models can be found in Table S1.

As previous studies have reported significant alterations in serum metabolite concentrations as a result of long-term (> 5 years) storage at − 80 °C^38,39^, we investigated whether biobanked samples could be used for model development. To this end, we used RRMS and SPMS samples obtained from the Welsh Neuroscience Research Tissue Bank which were stored at − 80 °C for between 1 and 10 years (Table S2). OPLS-DA models built using samples stored for between 1 and 10 years resulted in a predictive accuracy of only 58%. Although this accuracy is significantly greater than expected by random chance, as confirmed by permutation testing, this does not represent sufficient predictive accuracy for use in a clinical setting. As none of the potential demographic and clinical confounders investigated significantly impacted the metabolite biomarkers identified (Fig. S2), the decrease in diagnostic accuracy observed using the biobanked samples is not a result of differences between the cohorts but rather a result of long-term storage of the samples. Indeed, significant alterations in metabolite concentrations were observed in samples stored at − 80 °C for > 5 years compared to those stored for ≤ 5 years (Fig. S4). Furthermore, when only biobanked samples stored for ≤ 5 years were used for diagnostic test development, the accuracy improved to 66%. In contrast, our *well-validated* OPLS-DA model (Fig. 2, trained using optimal samples) was still able to diagnose (RRMS vs. SPMS) biobanked samples up to 5 years old with an accuracy of 66%, although the accuracy drops to 39% when predicting the diagnosis of samples stored for > 5 years. These results confirm that biobanked samples can be used for metabolite biomarker discovery in a research setting (although samples collected under optimal conditions should be used for clinical test development).

## Discussion

In this study, using an improved metabolomics algorithm on a prospective cohort of RRMS and SPMS patients, we were able to stage MS with 91% accuracy and the metabolite biomarkers identified agree with those in our previous work. We also introduced commonly encountered sample-handling variations to ‘stress-test’ our metabolomics algorithm and observed only a modest decrease in accuracy. Our current study supports the clinical applicability of this metabolomics test, by (1) further validating our results on an independent cohort, (2) confirming that the identified metabolite biomarkers remain discriminatory despite variations in sample-handling, and, thus, demonstrating that (3) samples collected prospectively from a ‘real-world’ setting are accurately diagnosed by our models.

The transition from RRMS to SPMS is phenotypically gradual and clinicians often diagnose SPMS much later (the mean period of diagnostic uncertainty was 2.9 years in one study) when there is clear sustained accumulation of disability^1^. Therefore, the diagnosis of SPMS is retrospective and further aggravated by a lack of consensus on clinical definitions^31^. As the paradigm of MS treatment shifts towards halting progression instead of merely reducing relapses, there is an urgent need for molecular biomarkers to objectively confirm SPMS and to monitor disease progression. Our approach has the potential to identify the onset of progression for clinical management purposes, as well as provide robust inclusion criteria for entry into SPMS clinical trials. Moreover, the use of serum allows serial samples to be taken for prospective monitoring of both the onset and rate of progression. Although we would recommend that blood samples are processed within 30 min of collection, immediately aliquoted, and stored at − 80 °C until analysis for model development and validation, accurate results are still obtained using samples collected in a clinical setting. Furthermore, samples stored for > 5 years can still provide significant and useful results for biomarker discovery in a research setting, although care should be taken as serum metabolite concentrations may change.

While the principal advantage of our multivariate analysis is to identify disease-specific metabolic signatures (i.e. combination of all metabolites), we were also able to discern specific discriminatory metabolites distinguishing RRMS and SPMS; higher lipoproteins, choline, 3-hydroxybutyrate in RRMS, and higher glucose and N-acetylated glycoproteins/glycolipids in SPMS. These are remarkably consistent (in terms of their importance and direction) across our current and previous cohorts, and may provide further clues to disentangle the neuroinflammatory and neurodegenerative processes that are present in the two MS disease phases, though to varying degrees. Indeed, recent NMR lipidomics studies have reported on the differences in lipoprotein subclasses and their immunomodulatory functions between RRMS, SPMS and age-gender matched healthy controls^40,41^. Our findings of higher 3-hydroxybutyrate and lower glucose in RRMS patients also parallel the observations of another NMR metabolomics study comparing MS patients (84% of whom were RRMS) and healthy controls^7^. Taking these findings in totality, these metabolites could provide inference to the pro-inflammatory state within RRMS. While there have been no studies reporting differences in blood choline levels between RRMS and progressive MS patients, ^1^H NMR studies have found increased choline (in both CSF and blood) in RRMS with respect to healthy controls as well as to non-MS controls^7,42^. Our finding of higher serum choline in RRMS may suggest higher myelin turnover (increased myelin breakdown and/or failure to synthesise myelin), as demonstrated by brain magnetic resonance spectroscopy (MRS) studies showing higher choline levels in RRMS patients compared to controls^43–45^.

We acknowledge that the remarkable accuracy achieved in distinguishing RRMS from SPMS in this study could be in part due to recruiting patient groups that are phenotypically more distinct along the continuum of MS disease course. However, patients with unequivocal SPMS status (and indeed RRMS) were required to allow us to construct the most discriminatory models and to use these to explore the accuracy changes arising from protocol variations. Furthermore, we did not find any potential confounders accounting for the metabolic differences between RRMS and SPMS patients despite extensive analyses. In particular, there was no association between any of the identified metabolites and age. Approximately 50–60% of RRMS patients will go on to develop SPMS about 20 years after disease onset^46,47^, therefore the older age in the SPMS group is a natural corollary rather than a bias in patient recruitment. There were no strong correlations of any of the top discriminatory metabolites with age, and indeed of the individual metabolite ‘bins’ with age (the highest R^2^ was 0.22). Stratification of our study cohort into 2 age groups, < 50 years and ≥ 50 years old, revealed no clustering on the OPLS-DA scores plot (Fig. S2A). In particular, RRMS patients who were ≥ 50 years old were evenly distributed throughout the RRMS cluster. These 2 approaches suggest that age is not a confounding factor in the metabolic distinction, both in terms of the individual discriminatory metabolites as well as the global metabolic profile, between these 2 MS stages. We also recognise the relatively small cohort size in our study as a possible limitation. However, the results presented reproduce our previous published results on independent patients adding substantial to weight to the validity of the biomarkers identified^13,14^. It should be noted that this is still the second largest cohort to be studied by serum NMR metabolomics^13^ and the third largest cohort with a robust and well-defined sample-handling protocol to investigate metabolomic differences between RRMS and SPMS by any metabolomics modality^18^. Other studies with larger cohorts have been performed using mass spectrometry methods, but these studies did not specify their sample-handling protocol, which is likely to vary due to the inclusion of samples from multiple centres and biobanks^16,17^. Furthermore, while these studies investigated larger cohorts overall, the number of SPMS patients in both reports is lower than presented here. Thus, this study represents, to date, the only simultaneous comparison of rigorously controlled sample-handling procedures from samples from a single blood draw, in a patient population.

## Conclusion

There is an unmet need for biofluid markers to differentiate these two phases of MS. Our study showed that RRMS and SPMS can be distinguished with high accuracy using serum metabolomics, and the accuracy our RRMS vs. SPMS test remained robust to variations in sample-handling encountered in the clinics. Future work will determine how the *well-validated* model performs on a large prospective cohort of patients that includes patients suspected to be transitioning to SPMS. Serial samples from these ‘transitional’ patients coupled with detailed clinical follow-up will determine the diagnostic accuracy of the test in a clinical setting, and these samples could also be used to model progression to allow predictive models to be constructed.

## Supplementary information

Supplementary Information.

## Data Availability

The metabolomics and clinical datasets, as well as supporting data generated and analysed during the current study are available to any qualified investigator from the corresponding author on reasonable request. Raw data files can be accessed through the Metabolights open access database (Study ID MTBLS1712).

## Abbreviations

ARR: Annualised relapse rate
AU: Arbitrary units
BMI: Body mass index
COSY: Correlation spectroscopy
CPMG: Carr–Purcell–Meiboom–Gill
DMT: Disease-modifying therapies
EDSS: Expanded disability status scale
MRI: Magnetic resonance imaging
MRS: Magnetic resonance spectroscopy
MS: Multiple sclerosis
NMR: Nuclear magnetic resonance
OPLS-DA: Orthogonal partial-least square discriminant analysis
ppm: Parts per million
RRMS: Relapsing–remitting multiple sclerosis
SPMS: Secondary progressive multiple sclerosis
TOCSY: Total correlation spectroscopy
VIP: Variable importance in projection

## References

[1] Katz Sand I, Krieger S, Farrell C, Miller AE (2014). Diagnostic uncertainty during the transition to secondary progressive multiple sclerosis. Multiple Scler..

[2] Plantone D, De Angelis F, Doshi A, Chataway J (2016). Secondary progressive multiple sclerosis: definition and measurement. CNS Drugs.

[3] Lublin FD (2014). Defining the clinical course of multiple sclerosis: the 2013 revisions. Neurology.

[4] Wishart DS (2016). Emerging applications of metabolomics in drug discovery and precision medicine. Nat. Rev. Drug Discov..

[5] Patti GJ, Yanes O, Siuzdak G (2012). Innovation: metabolomics: the apogee of the omics trilogy. Nat. Rev. Mol. Cell Biol..

[6] Sylvestre DA, Slupsky CM, Aviv RI, Swardfager W, Taha AY (2020). Untargeted metabolomic analysis of plasma from relapsing-remitting multiple sclerosis patients reveals changes in metabolites associated with structural changes in brain. Brain Res..

[7] Cocco E (2016). (1)H-NMR analysis provides a metabolomic profile of patients with multiple sclerosis. Neurol. Neuroimmunol. Neuroinflamm..

[8] Mehrpour M, Kyani A, Tafazzoli M, Fathi F, Joghataie MT (2013). A metabonomics investigation of multiple sclerosis by nuclear magnetic resonance. Magn. Reson. Chem..

[9] Kasakin MF (2019). Targeted metabolomics approach for identification of relapsing-remitting multiple sclerosis markers and evaluation of diagnostic models. MedChemComm.

[10] Andersen SL (2019). Metabolome-based signature of disease pathology in MS. Multiple Scler. Relat. Disord..

[11] Poddighe S (2017). Metabolomic analysis identifies altered metabolic pathways in multiple sclerosis. Int. J. Biochem. Cell Biol..

[12] Villoslada P (2017). Metabolomic signatures associated with disease severity in multiple sclerosis. Neurol. Neuroimmunol. Neuroinflamm..

[13] Dickens AM (2014). A type 2 biomarker separates relapsing-remitting from secondary progressive multiple sclerosis. Neurology.

[14] Aguilar JA (2019). Reliable, high-quality suppression of NMR signals arising from water and macromolecules: application to bio-fluid analysis. Analyst.

[15] Herman S (2018). Integration of magnetic resonance imaging and protein and metabolite CSF measurements to enable early diagnosis of secondary progressive multiple sclerosis. Theranostics.

[16] Lim CK (2017). Kynurenine pathway metabolomics predicts and provides mechanistic insight into multiple sclerosis progression. Sci. Rep..

[17] Senanayake VK, Jin W, Mochizuki A, Chitou B, Goodenowe DB (2015). Metabolic dysfunctions in multiple sclerosis: implications as to causation, early detection, and treatment, a case control study. BMC Neurol..

[18] Lazzarino G (2017). Serum compounds of energy metabolism impairment are related to disability, disease course and neuroimaging in multiple sclerosis. Mol. Neurobiol..

[19] Barbour C (2017). Molecular-based diagnosis of multiple sclerosis and its progressive stage. Ann. Neurol..

[20] Anton G (2015). Pre-analytical sample quality: metabolite ratios as an intrinsic marker for prolonged room temperature exposure of serum samples. PLoS ONE.

[21] Yin P (2013). Preanalytical aspects and sample quality assessment in metabolomics studies of human blood. Clin. Chem..

[22] Jobard E (2016). A systematic evaluation of blood serum and plasma pre-analytics for metabolomics cohort studies. Int. J. Mol. Sci..

[23] Teahan O (2006). Impact of analytical bias in metabonomic studies of human blood serum and plasma. Anal. Chem..

[24] Fliniaux O (2011). Influence of common preanalytical variations on the metabolic profile of serum samples in biobanks. J. Biomol. NMR.

[25] Pinto J (2014). Human plasma stability during handling and storage: impact on NMR metabolomics. Analyst.

[26] Bernini P (2011). Standard operating procedures for pre-analytical handling of blood and urine for metabolomic studies and biobanks. J. Biomol. NMR.

[27] Hernandes VV, Barbas C, Dudzik D (2017). A review of blood sample handling and pre-processing for metabolomics studies. Electrophoresis.

[28] Bervoets L (2015). Influence of preanalytical sampling conditions on the 1 H NMR metabolic profile of human blood plasma and introduction of the Standard PREanalytical Code used in biobanking. Metabolomics.

[29] Thompson AJ (2018). Diagnosis of multiple sclerosis: 2017 revisions of the McDonald criteria. Lancet Neurol..

[30] Kremenchutzky M, Rice GP, Baskerville J, Wingerchuk DM, Ebers GC (2006). The natural history of multiple sclerosis: a geographically based study 9: observations on the progressive phase of the disease. Brain.

[31] Lorscheider J (2016). Defining secondary progressive multiple sclerosis. Brain.

[32] Kurtzke JF (1983). Rating neurologic impairment in multiple sclerosis: an expanded disability status scale (EDSS). Neurology.

[33] Yin P, Lehmann R, Xu G (2015). Effects of pre-analytical processes on blood samples used in metabolomics studies. Anal. Bioanal. Chem..

[34] Jurynczyk M (2017). Metabolomics reveals distinct, antibody-independent, molecular signatures of MS, AQP4-antibody and MOG-antibody disease. Acta Neuropathol. Commun..

[35] Wishart DS (2017). HMDB 4.0: the human metabolome database for 2018. Nucleic Acids Res..

[36] R Core Team (R Foundation for Statistical Computing, Austria). *R: A Language and Environment for Statistical Computing*. (2013).

[37] Thevenot EA, Roux A, Xu Y, Ezan E, Junot C (2015). Analysis of the human adult urinary metabolome variations with age, body mass index, and gender by implementing a comprehensive workflow for univariate and OPLS statistical analyses. J. Proteome Res..

[38] Yang W (2013). Liquid chromatography-tandem mass spectrometry-based plasma metabonomics delineate the effect of metabolites' stability on reliability of potential biomarkers. Anal. Chem..

[39] Haid M (2018). Long-term stability of human plasma metabolites during storage at -80 degrees C. J. Proteome Res..

[40] Jorissen W (2017). Relapsing-remitting multiple sclerosis patients display an altered lipoprotein profile with dysfunctional HDL. Sci. Rep..

[41] Gafson AR (2018). Lipoprotein markers associated with disability from multiple sclerosis. Sci. Rep..

[42] Reinke SN (2014). Metabolomic profiling in multiple sclerosis: insights into biomarkers and pathogenesis. Multiple Scler..

[43] Kirov II (2009). MR spectroscopy indicates diffuse multiple sclerosis activity during remission. J. Neurol. Neurosurg. Psychiatry.

[44] Inglese M (2003). Diffusely elevated cerebral choline and creatine in relapsing-remitting multiple sclerosis. Magn. Reson. Med..

[45] Tartaglia MC (2002). Choline is increased in pre-lesional normal appearing white matter in multiple sclerosis. J. Neurol..

[46] Tremlett H, Yinshan Z, Devonshire V (2008). Natural history of secondary-progressive multiple sclerosis. Multiple Scler..

[47] Vukusic S, Confavreux C (2003). Prognostic factors for progression of disability in the secondary progressive phase of multiple sclerosis. J. Neurol. Sci..

