# Supplementary material for: A blood-based metabolomics test to distinguish relapsing–remitting and secondary progressive multiple sclerosis: addressing practical considerations for clinical application
Source: Sci Rep. 2020 Jul 24;10:12381. doi: 10.1038/s41598-020-69119-3 (PMC7381627; doi:10.1038/s41598-020-69119-3)
Supplement: Supplementary file 1 — Supplementary Information. [file 41598_2020_69119_MOESM1_ESM.docx]

**A blood-based metabolomics test to distinguish relapsing-remitting and secondary progressive multiple sclerosis: addressing practical considerations for clinical application**

**Authors**

Tianrong Yeo,^a,b^, Megan Sealey^a^, Yifan Zhou^a^, Luisa Saldana^c^, Samantha Loveless^d^, Timothy D.W. Claridge^e^, Neil Robertson^d^, Gabriele DeLuca^c^, Jacqueline Palace^c^, Daniel C. Anthony^a^, Fay Probert*^a^

*Corresponding author

**Affiliations**

^a^Department of Pharmacology, University of Oxford, Mansfield Road, Oxford OX1 3QT, UK.

^b^Department of Neurology, National Neuroscience Institute, 11 Jalan Tan Tock Seng, Singapore 308433, Singapore.

^c^Nuffield Department of Clinical Neurosciences, Level 6, West Wing, John Radcliffe Hospital, University of Oxford, Headley Way, Oxford OX3 9DU, UK.

^d^Division of Psychological Medicine and Clinical Neuroscience, School of Medicine, University Hospital of Wales, Cardiff University, Cardiff, CF14 4XN.

^e^Chemistry Research Laboratory, Department of Chemistry, University of Oxford, Mansfield Road, Oxford OX1 3TA, UK.

**Table S1.** Summary indices of OPLS-DA models using samples collected within and across the different sample-handling protocols.

|  | **Train: *Optimised* protocol**  **Test: *Optimised* protocol** | **Train: *Freeze-thaw* protocol**  **Test: *Freeze-thaw* protocol** | **Train: *120 minutes* protocol**  **Test: *120 minutes* protocol** | **Train: *240 minutes* protocol**  **Test: *240 minutes* protocol** |
| --- | --- | --- | --- | --- |
| Accuracy ± SD (%) | 91.0 ± 3.0 | 89.6 ± 3.3 | 81.7 ± 3.5 | 84.9 ± 3.2 |
| Sensitivity ± SD (%) | 92.3 ± 4.4 | 87.2 ± 4.5 | 83.8 ± 5.4 | 88.5 ± 4.6 |
| Specificity ± SD (%) | 91.6 ± 3.7 | 94.1 ± 4.0 | 83.4 **±** 4.0 | 84.1 ± 4.5 |
|  |  |  |  |  |
|  |  |  |  |  |
|  | **Train: *Optimised* protocol**  **Test: *Freeze-thaw* protocol** | **Train: *Optimised* protocol**  **Test: *120 minutes* protocol** | **Train: *Optimised* protocol**  **Test: *240 minutes* protocol** | – |
| Accuracy ± SD (%) | 85.5 ± 3.8 | 85.9 ± 3.1 | 88.0 ± 3.0 | – |
| Sensitivity ± SD (%) | 91.1 ± 4.1 | 78.5 ± 5.9 | 83.4 ± 5.4 | – |
| Specificity ± SD (%) | 80.6 ± 6.6 | 94.0 ± 4.1 | 93.0 ± 5.3 | – |
|  |  |  |  |  |
|  |  |  |  |  |
|  | **Combination 1**  **Train: *Optimised* + *Freeze-thaw* protocol**  **Test: *Optimised* + *Freeze-thaw* protocol** | **Combination 2**  **Train: *Optimised* + *120 minutes* protocol**  **Test: *Optimised* + *120 minutes* protocol** | **Combination 3**  **Train: *Optimised* + *240 minutes* protocol**  **Test: *Optimised* + *240 minutes* protocol** | **Combination 4**  **Train: *Optimised* + freeze-thaw + 120 minutes + *240 minutes* protocol**  **Test:  *Optimised* + freeze-thaw + 120 minutes + *240 minutes* protocol** |
| Accuracy ± SD (%) | 87.7 ± 3.9 | 86.2 ± 4.1 | 87.1 ± 5.0 | 71.9 ± 6.3 |
| Sensitivity ± SD (%) | 87.9 ± 5.9 | 86.0 ± 5.1 | 86.3 ± 6.1 | 71.8 ± 9.8 |
| Specificity ± SD (%) | 88.3 ± 6.0 | 86.7 ± 6.9 | 88.6 ± 7.4 | 72.6 ± 9.9 |

**Table S2.** Demographic and clinical data of the Welsh Neuroscience Research Tissue Bank cohort.

|  | **RRMS (n = 30)** | **SPMS (n = 50)** |
| --- | --- | --- |
| Age (years,) mean ± SD | 40.0 ± 10.2 | 50.4 ± 10.4 |
| Female, no. (%) | 19 (63.3) | 26 (52.0) |
| EDSS, mean ± SD | 5.1 ± 0.8 | 6.0 ± 0.9 |
| Sample storage duration (years), mean ± SD | 6.5 ± 3.0 | 8.0 ± 2.0 |

**Fig. S1**

**Fig. S1.** VIP ranking plot identifies the top discriminatory metabolites from the *optimised* protocol. The dashed line indicates the VIP score cutoff at 1.48.

VIP: variable importance in projection.

**Fig. S2**

**Fig. S2.** Scores plots of the *well-validated* RRMS (circle) and SPMS (diamond) OPLS-DA model shaded according to potential confounding factors. In each instance, the potential confounder of interest (age, disease duration, presence of relapses, disability status, DMT use, comorbidity status, BMI, alcohol intake, and prandial status) is evenly distributed throughout each disease classification.

BMI: body mass index; EDSS: Expanded Disability Status Scale; DMT: disease modifying therapy; OPLS-DA: orthogonal partial-least square discriminant analysis; RRMS: relapsing-remitting MS; SPMS: secondary progressive MS.

Fasted is defined as ≥ 6 hours since last meal, while fed is defined as < 2 hours since last meal.

**Fig. S3**

**Fig. S3.** VIP ranking of top metabolite ‘bins’ across the models trained on sub-optimal samples.

VIP: variable importance in projection.

**Fig. S4**

**Fig. S4.** Long-term (> 5 years) storage at −80 °C results in significant changes to glucose, lactate and lipoprotein metabolite concentrations.
